# Cognition About the Creative Process – Interview With Dr Andrew P. Allen

**DOI:** 10.5964/ejop.v12i4.1323

**Published:** 2016-11-18

**Authors:** Andrew P. Allen, Lynda Loughnane

**Affiliations:** a University College Cork, Cork, Ireland; b Crawford College of Art and Design, CIT, Cork, Ireland

**Keywords:** creativity, consciousness, learning, phenomenology, mindfulness

## Abstract

What is the relationship between the creative process and cognition and perception? Lynda Loughnane, a master’s student in Art and Process in Crawford College of Art and Design, Cork, Ireland interviewed Dr Andrew P. Allen about the subject. Areas covered include mindfulness, Type 1 and Type 2 thinking, stage theories of creativity, engagement with the art process and the artwork, phenomenology and consciousness with and without self report. The interview was constructed to cover a wide range of subject matter, so as to gather as much information as possible in layman's language about the cognitive process in relation to creativity and interaction with art.

**Lynda Loughnane:** First of all thank you very much for agreeing to meet me. I have been talking to a number of professionals in different fields to get as wide a view point as possible in the areas of light, perception and cognition. I will then use this information to inform my creative process. To begin, you mentioned Type 1 and Type 2 thinking in relation to creative thinking in your blog. Can you talk a bit more about this please?

**Andrew P. Allen:** Type 1 and Type 2 thinking are broad terms to describe levels of thinking. Type 1 thinking is thought to be quite fast or automatic, or if not automatic, at least heuristic. Type 2 thinking is considered more step by step and logical; often tied to more formal styles of thinking such as logic or mathematics. It is probably better to think of this as a continuum rather than two completely distinct boxes. About five years ago I wrote a paper which was partly inspired by one cognitive psychologist who described various aspects of cognition and different ways of thinking about Type 1 and Type 2 (Allen & Thomas, 2011). Steven Sloman (1996) described how creative thinking and imagination would be archetypical of Type 1 thinking, so automatic. In my review paper, I argued that both types of thinking are quite important to creative thinking and I linked it in with stage theories of the creative process. Stage theory goes back to Wallas in the 1920s (Wallas, 1926). It states that one would have, within the creative process, the initial stage of conceptualisation of what you want to do creatively. Then the person often reaches an impasse where they may have to stop for a while, which is a stage of problem solving or a subtype of creative thinking. There is an incubation stage where you leave the problem aside for a while. Perhaps they then get inspired later on by some other thing that they encounter in their life. They have insight where they start having new ideas. Beyond that, once they have these insights, there is a lot of leg work within the creative process generally. In fairness to Sloman, he did include verification as under the Type 2 thinking which has been included in stage theories of creativity as part of the creative process. The overarching idea behind this paper I was writing was to highlight how both types of thinking can be quite important to the creative process and how the differing roles during different phases of the creative process are important as well.

[Contrib contnr1]**Lynda Loughnane:** I absolutely agree with you, speaking from an artist's point of view. Of course Type 1 is that moment of 'Oh, I am inspired, I have an idea' but you also go through a lot of Stage 2! You slog through it ... to put it in layman terms!

Do you think that the heuristic Type 1 thinking is similar to affect? What I mean by affect is that gut reaction you have to something.

**Andrew P. Allen:** Let's look at the work of Paul Slovic (e.g. Slovic, Finucane, Peters, & MacGregor, 2002). He describes affect as being a form of heuristic thinking - that you can use your gut reaction, if you like, as a source of information about how you wish to deal with a particular aspect of your life or a decision that you want to make. For example, if you have a negative affect toward something; even if you are not able to articulate exactly why you feel negatively about it, you might, nonetheless take that affect and have a kind of heuristic reaction. This is a type of decision making rule, whereby if you have a negative response towards something or a negative affect, then you are going to ignore this option within your decision making plan. Whereas, if you have an initial positive affect, you might not choose that option immediately but you might seek more information about it rather than writing it off, which is quite often the case if you have the negative affective response. I certainly think affect and heuristics can tie together.

**Lynda Loughnane:** With my work, I would like to hope that the two types of thinking would be sparked in the viewer. First, the gut reaction, then the 'not quite sure what I am looking at but I want to figure out what it is' reaction. Do you think it is possible for an artwork to do this?

**Andrew P. Allen**: In terms of the phrasing of the question, you've asked if the artwork itself can do it: Let's start with the viewers themselves responding to it. A lot of people who move through busy galleries will only have so much time to engage in any particular piece. If something hits them at a gut level, they can then begin to think of it on a more ‘step by step’ level. If someone wishes to engage in this verification, if you like, as a consumer rather than a producer of a creative work - they can stretch the thinking out in a more step by step level of appraisal. That doesn't mean that that won't continue to be influenced by, for example, their initial negative response. Step by step thinking isn't always completely objective. It can still be coloured by their initial intuition about the piece of art. I should say when I talk about Type 1 and Type 2 thinking, even in themselves, there are differing theories about how these two forms of thinking interact in general. For example whether Type 2 can override Type 1 or, to some extent, that Type 1 comes back or can change in response to Type 2 and so forth. There is still a lot of debate in general about how these two forms of thinking can interact (e.g. Evans, 2007), particularly given that there is a probably a continuum between conscious processing involving some decision making and then something which is very logical and formal.

[Contrib contnr2]**Lynda Loughnane:** You mentioned consciousness with and without self report in your blog. Can you explain this a little bit further?

**Andrew P. Allen**: Well, typically if something is unconscious we can say that it’s Type 1, but consciousness can be defined in different ways. You can think of it as being at a simple level of being awake or having some basic awareness of one's environment. Then there is a more complex level, when you think of self-awareness, which is often what people talk about with consciousness. And then there is what David Chalmers (1995) has called the 'hard problem of consciousness'. This is the qualia, the subjective sense of what it is like to have a particular experience. You find with the 'hard problem of consciousness', which is quite private, is that it’s something we all presumably experience ourselves, but we struggle in terms of seeing what it is like for someone else. For example, we could speculate about differences between us in terms of our experience or who we are, but we cannot know exactly what it is like to be two different people, although we can self-report. The hardest problems within that is trying to think about other systems that can process information and what their qualia might be like. Indeed whether they even have a subjective sense of what it is like to be themselves, so for example, with small infants, non-human animals, and artificial intelligence and so on, self-report simply isn't available. In other words methodologies have to be worked out which don't require self-report.

With self report you could use techniques that try, in terms of neuroscience, to look at which areas of the brain are activated by a stimulus when you are conscious of it, versus when you are not conscious of it. Experimenters want to control it quite carefully so the ideal situation would be to have a person exposed, at a sensory level, to the same stimulus but to have them conscious of it or not conscious of it. This can be achieved using a method known as interocular suppression, in which you present different images to the two eyes. If you have a given stimulus presented to one eye, consciousness can come and go when you have, for example, flash suppression coming into the other eye so you have constant sensory input with altering subjective consciousness. You then get the person to self-report whether they are conscious or not conscious of a particular thing and investigate which sensory areas of the brain are being activated when a person has a subjective sense of consciousness versus when they do not. That is one potential way of looking at self reporting consciousness.

**Lynda Loughnane:** Do you think the fact that they are reporting on it changes their consciousness?

**Andrew P. Allen:** Potentially, yes. There was an interesting review last year (Tsuchiya, Wilke, Frässle, & Lamme, 2015) which compared the two methods and one issue they brought up was that reporting consciousness could potentially alter it. I suppose it depends on what kind of complexity you are talking about in terms of report. In terms of interocular suppression paradigm, you could ask the person if they aware of something or not; a simple yes or no kind of question. But it is a different matter if you want people to talk about self report on what the actual qualia was like. I'm sure you can imagine that even if you were James Joyce, you are going to be limited in terms of your stream of consciousness and the amount of sensory information you take in, which is complex. Even in terms of the stuff you are consciously aware of, it is incredibly difficult to give a full report on what it is that actually got through. So the process of self reporting can be quite fraught.

**Lynda Loughnane**: The next question is about phenomenology. Maurice Merleau-Ponty was a proponent of phenomenology and he questioned Cartesian perspectivism (e.g. Merleau-Ponty, 1962) which felt reality existed only in the brain. I was wondering what was your thoughts on these two ways of viewing the world.

**Andrew P. Allen**: Well, the way you said that verbally is 'inside the brain' and the question you have written (*in a questionnaire given in advance of the interview*) was 'inside the mind'. What you did there would be describe the identity theory, which considers that the mind is what the brain does - that the brain and the mind are one. People think increasingly of the mind as being embodied which I suppose is what Merleau-Ponty was quite keen on. We do cognition in the head but it is also involved in the way in which we use the rest of our bodies, including guts (Dinan, Stilling, Stanton, & Cryan, 2015)! If you think about mind in terms of information processing that humans engage in, there's a continuum between what happens within the skull, and in broader terms what is happening with different nervous systems which are distributed throughout the body. For example how we respond to the autonomic system, the fight or flight response; obviously our brain can set off signals but we can respond bodily as well, with things such as with increased heart rate or sweating. I think the cognition process can certainly extend beyond the brain, to bodily events and it can also extend into the information processing tools that we use in our everyday lives to augment our memories and attentions and so forth.

Sticking with what is happening internally, within our brains, we have a certain interpretation of things that are happening in our environment. For example, the objective information that might be in a book: We can, even in a very dry information processing sense, come to different interpretations of what we are reading. Our interpretation, which comes from how you process information, can then be used as an extra source of information in which to parse quite complex information into simpler things, which can then be used for heuristic decision making.

**Lynda Loughnane:** You had some very interesting blog reports about mindfulness, which states it has the ability to bring the attention to the present moment. Do you think it is possible to interact with a piece of art on its own terms, or do we always bring our own past projected self to this interaction?

**Andrew P. Allen:** Within attention people talk about 'top down' attention which is driven by things such as previous experience or what our motivations are, like the baggage we bring to our thinking about things, versus 'bottom up' thinking, which can be more driven by sensory input. I understand why people might think that we always bring our past or projected self into an interaction with art. Although we might have certain sensory inputs which we don't control, for example, we turn one way in the gallery versus that way; our own sensory inputs are often motivated by our own decisions, in terms of whether we decide to look at particular forms of art or if we decide to pursue this particular genre, to consume this or that form of artistic input. The real question here is can we escape from that and to what extent. If we see something that for us is really quite novel, something that is quite new, then I think our cognitive baggage, within a few seconds, will have to have some impact upon that. I do think that within a certain amount of time, you are going to have to think about your past interpretations of other pieces of art and how it relates. At least in some limited sense, you can try to approach a piece of art on its own terms and try to have some kind of 'bottom up' attention, particularly with a complex piece of work; seeing the actual content in terms of what it is trying to portray. I think it is significantly difficult to try to break away from our past psychological baggage when consuming art but I think in some limited sense you can try to approach it on its own terms.

You mentioned mindfulness as well in terms of bringing the attention to the present moment so yes, perhaps mindfulness is one potential way of dealing with current experience. With mindfulness, especially in relation to stress reduction, you focus on things like your own body, your own breath, and your own sensations of your immediate body. I see no reason why it couldn't be used to focus your awareness on things that are external to you such as a piece of art to control your attention in such a way as to focus on the particular piece that you are trying to understand. If your mind wanders to previous pieces of art that you have seen or perhaps an emotional response, then using mindfulness techniques, you might be able to gently redirect your attention back to the piece of art itself, on its own terms without a runaway stream of consciousness about something else you have been ruminating on in the past.

**Lynda Loughnane:** The fact that the piece of art is produced by somebody else who has their own consciousness and their own set of agendas behind that piece of art, makes it quite hard to be with the art piece just as an object and not feel the draw of the person behind the art.

What are your thoughts on how the creative process affects the behaviour of a person and does the act of engaging in a creative process, or being exposed to a piece of art change the way the person 'sees' their reality? There are two very distinct questions within that one question; one is interacting with art and one is viewing art.

**Andrew P. Allen:** In terms of cognition generally, I mentioned earlier we can think of cognition as extended in terms of how we think, which does not simply draw on what is going on in our brains - we can interact with tools around us to augment cognition. Vlad Glăveanu (2014) has talked about this in more depth. Say you are a sculptor, in order to create an assemblage sculpture, you might be interacting with the different components of the piece. You may be turning them over in an attempt to perceive them from different perspectives and to ascertain how they can interact with the other components that make up the whole. In that sense, the process of going through, perhaps a Type 2 process, and how you respond to that on an affective level, can bring about different aspects of cognition. I think you might see the different components that you have chosen to study in a greater depth both in terms of their basic physical characteristics such as their mass, shape and form and your response to them, as well gauging the response of a potential audience. That would impact on how you see what you are interacting with. If that then increases your mindfulness once you leave the studio, it could impact on the way you see things around you, or not, depending to what extent it has a hangover effect when you are no longer doing it.

In terms of being exposed to art, from a consumer point of view, that is perhaps a trickier question because it may not be extended to the same level, unless the artist is actually encouraging you to poke about the sculpture. Perhaps it is not as strong or striking as the active process of creating art itself, but that depends to what extent the person is actually engaging with the art work. If they are actively engaged, then I imagine a lot of the same mechanisms would apply.

**Lynda Loughnane:** Do you think practices such as meditation or other activities which activate what is sometimes called the 'Inner Eye' such as chanting or praying have an effect on the cognitive or perceptive abilities of practitioners? What happens at a neurological level with such activities, in relation in particularly to sight, as this is my area of interest ... do practitioners see or perceive differently?

**Andrew P. Allen**: In terms of the neuroscience, Michael Posner is a major person in the field of attention. He and his colleagues recently wrote an interesting review about neuroscience and mindfulness (Tang, Hölzel, & Posner, 2015). I’m not sure if mindfulness having a major impact on the brain in areas such as the occipital lobe that you might associate with sensory processing or visual information so I don’t know to what extent it would impact on sight, for example. It can impact upon attention, certainly. There is evidence that mindfulness can alter activity in the anterior cingulate cortex, which is associated with more complex cognition such as conflict monitoring. For example, if you are responding to a task where you have to attend to multiple aspects of different stimuli. Then with a particular trial, you have to respond to one particular aspect of that stimuli, but there is another aspect that makes you want to respond in a different way, you then have to monitor that kind of conflict. It seems mindfulness can help with that as well as with sustained attention, which I am particularly interested in. This is the ability to remain processing a particular steam of information without being distracted, essentially. There is evidence that the frontal cortex activity can be enhanced by mindfulness practice. It is intuitive that this would be the case, because when you are engaged in mindfulness practice, you are trying to sustain your attention on sensations within you. For example, if you are doing a body scan, you specifically want your attention on your breath. If you then start to think about what you are going to have for your lunch, that is a distraction, so you try to gently bring your attention back to your breath. In this way mindfulness is a practice of sustained attention. In that sense it is encouraging to see that regions of the brain associated with these kind of processes, can be enhanced by engaging in mindfulness practice.

**Lynda Loughnane:** Something I am curious about is if meditation or mindfulness can affect areas of the brain such as the occipital lobe and if these areas can in some way be activated through intense introspection... Perhaps not but it is something I wonder about.

**Andrew P. Allen:** It is possible. Research on mindfulness is sometimes done over an eight week course to see affects within people. You could base research on improvements in their attention, but that might not be an intense expert level of intense introspection. What is interesting about mindfulness is that it can be used in stress reduction. Mindfulness based stress reduction is quite a widely practiced form of mindfulness. One of the areas I am interested in is the interaction between stress and cognition. For example, with conditions of chronic stress (e.g. caring for a relative with dementia) you might anticipate that over time this can have a negative impact on the brain through, for example, excessive cortisol activation. If there are ways of attenuating stress, it might have a positive knock-on effect on cognition. Indeed, there is evidence that interventions to reduce stress can improve cognitive performance in dementia caregivers (c.f. review by Allen et al., in press).

**Lynda Loughnane:** Speaking from an artist's point of view, you can get so involved in being present with the creative process that involvement with an art activity can be hugely stress relieving; you are so focused on the present moment that nothing else permeates the process. It's an effective tool for mindfulness, I personally find.

**Andrew P. Allen:** Csikszentmihalyi (1996) talks about 'flow'. It seems to be quite a mindful kind of state where you become very absorbed in a particular activity. This idea of 'flow' came from the study of the psychology of creativity. It seems to be an archetypical part of creative work, an archetypical example of flow state.

**Lynda Loughnane:** When you get into a state of flow it's an amazing place to be! Thank you again very much, that was very informative and interesting. And I just want to mention again that I really enjoy your blog.

**Andrew P. Allen:** Thank you!
